# Association Between Programed Cell Death-1 and CD4^+^ T Cell Alterations in Different Phases of Ischemic Stroke Patients

**DOI:** 10.3389/fncel.2018.00170

**Published:** 2018-06-22

**Authors:** Yi Zhang, Li Wei, Yupeng Du, Yirui Xie, Wei Wu, Yuan Yuan

**Affiliations:** ^1^Department of Laboratory Medicine, The First Affiliated Hospital, College of Medicine, Zhejiang University, Hangzhou, China; ^2^State Key Laboratory of Diagnostic and Treatment of Infectious Diseases, The First Affiliated Hospital, College of Medicine, Zhejiang University, Hangzhou, China; ^3^Department of Rehabilitation, The Third Affiliated Hospital, Zhejiang University of Traditional Chinese Medicine, Hangzhou, China; ^4^Department of Neurology, The First Affiliated Hospital, College of Medicine, Zhejiang University, Hangzhou, China

**Keywords:** ischemic stroke, T cells, Tim-3, PD-1, stroke-induced immunodepression (SIID)

## Abstract

**Objective**: We aimed to analyze alterations in T cell subgroups during different post-ischemic stroke (IS) phases to explore the possible mechanisms underlying stroke-induced immune depression (SIID).

**Methods**: Sixty-four IS patients who met the entry criteria were divided into three groups: an acute phase group, a sub-acute phase group and a stable phase group. Fourteen healthy individuals were selected as normal controls. The phenotype distribution of T cells in patient peripheral blood was analyzed, and the immune checkpoint receptors programed cell death-1 (PD-1) and T cell immunoglobulin and mucin domain 3 (Tim-3) were detected in different T cell phenotypes.

**Results**: Compared with the control group, the absolute number of CD4^+^ T cells and CD4^+^ T central memory (TCM) cells was significantly increased in the acute phase group but decreased in the sub-acute phase and stable phase groups compared with that in the acute phase group. PD-1 expression in CD4^+^ T cells in the stable phase group showed a significant increase compared with that in the acute phase group. The expression of PD-1 on CD4^+^ TCM cells and CD4^+^ T effector memory (TEM) cells showed significant decreases in the acute phase compared with control cells; however, in the sub-acute phase and the stable phase, PD-1 expression was significantly increased compared with that in the acute phase.

**Conclusions**: T cell dysfunction, especially CD4^+^ T cell dysfunction, occurred during different IS phases. PD-1 was highly expressed in CD4^+^ T cells of different phenotypes after the acute phase and was associated with alterations in CD4^+^ T cells. Particularly, PD-1 was negatively correlated with the absolute number of TCM cells among different CD4^+^ T cell phenotypes, which may be one of the possible mechanisms of SIID.

## Introduction

Stroke is a disease associated with high death and disability worldwide and results in permanent neurological damage to patients (Strong et al., 2007). Ischemic stroke (IS), which accounts for 80%–85% of stroke cases, is frequently induced by thromboembolic occlusion of the cerebral artery. In particular, infectious complications have been reported to occur in 23%–65% of stroke patients after stroke, which leads to poor recovery of patients (Chamorro et al., 2007).

The immune system is considered to play critical roles in patient outcomes following stroke, including in acute stroke events and long-term post-stroke recovery (Bravo-Alegria et al., 2017). In a mouse model, Prass et al. (2003) reported that stroke induced immunodeficiency, which increases susceptibility to bacterial infections. In a human study, impaired T cell responses and decreases in peripheral lymphocyte counts have been reported in stroke patients (Haeusler et al., 2008).

IS induces two immunological cascades: an autoimmune response to central nervous system (CNS) antigens that induces brain inflammation and stroke-induced immunodepression (SIID), which impairs resistance to bacterial infections, resulting in increased incidence of infection, particularly urinary tract infections and pneumonia (Vogelgesang and Dressel, 2011). However, the mechanisms leading to SIID remain unclear, which seriously affects prevention and control of infectious complications in the clinic.

T cell immunity is involved in IS. T cell, but not B cell, contributions to inflammatory and thrombogenic responses have been reported in mice IS (Yilmaz et al., 2006). Chamorro et al. (2005) reported that T cells shift from a Th1-type response to a Th2-type response post-stroke phase. Tregs have been reported to reduce the infarct volume in rats subjected to transient brain ischemia. Dolati et al. (2018) reported that increased Th17 cells and decreased Tregs might contribute to the pathogenesis of IS. Furthermore, accumulating evidence has shown that T cells play important roles in the stroke process (Ishibashi et al., 2002).

T cells are classified as naïve, effector, or memory cells according to the expression of CD45 isoforms and CCR7, a chemokine receptor that helps T cells home to lymph nodes (Ebert et al., 2005). Memory T cells in the context of persistent herpes virus infection contribute to relative control and immunosurveillance of active replication or viral reactivation (Torti and Oxenius, 2012). Effector T cells have a superior capacity to respond quickly to antigenic stimulation compared with naïve T cells (Sallusto and Lanzavecchia, 2009). However, the roles of effective/memory T cells in stroke currently remain unclear. Low numbers of T lymphocytes flow into the healthy brain; however, the role of these cells in stroke is not well understood but has increasingly attracted the attention of researchers (Gemechu and Bentivoglio, 2012).

Our research group previously reported that programed cell death-1 (PD-1) and T cell immunoglobulin and mucin domain 3 (Tim-3) are important cell surface markers that play key roles in immune responses. In addition, Zhao et al. (2011) reported that an increase in Tim-3 is positively correlated with IL-17 and TNF-α levels in IS. Ren et al. (2011) reported that the PD-1 pathway limited CNS inflammation and neurologic deficits in an animal stroke model. Therefore, we hypothesized that PD-1/Tim-3 may be associated with T cell alterations in stroke, which may be one of the mechanisms of SIID.

Therefore, to verify our hypothesis and explore the mechanisms underlying SIID, we investigated T cell alterations during different post-stroke phases and the correlations of PD-1 and Tim-3 with the T cell alterations.

## Materials and Methods

### Patients

Sixty-four IS patients were recruited from 10 June 2016 to 6 June 2017. IS was diagnosed based on the criteria issued by the World Health Organization Multinational Monitoring of Trends and Determinants in Cardiovascular Disease (WHO-MONICA) and verified by computed tomography (CT) or magnetic resonance imaging (MRI). The patients were divided into three groups based on the time post-stroke: an acute phase group (defined as within 24 h post-IS), sub-acute phase group (defined as 48 h to 10 days post-IS), and stable phase group (defined as 10–30 days post-IS). The following clinical data were recorded for the stroke subjects: age, gender, race, smoking status, alcohol use status, other disease status, the National Institutes of Health Stroke Scale (NIHSS) score and the mini-mental state examination (MMSE) score. Infection was defined as clinical symptoms of an infection, including fever, pyuria for urinary tract infection, productive cough, radiographic evidence of consolidation for pneumonia, and positive bacterial culture. The exclusion criteria were as follows: those with a history of cerebral hemeorrhage, encephalopyosis, transient ischemic attack, surgery or trauma within the last 3 months, hemeatology disorders, malignancy and congestive heart failure. Fourteen age- and sex-matched healthy individuals served as normal controls. Each subject or their guardians signed an informed consent. The study protocols were approved by the Ethical Committee of the First Affiliated Hospital, College of Medicine, Zhejiang University.

### Methods

Venous blood (5 ml) was collected from each subject in a fasting state between 08:00 am and 09:00 am on d2 after grouping. The total lymphocyte count in the whole blood was immediately detected after sample collection with an ABX Pentra DX120 system (Horiba Medical, Montpellier, France).

The T cells, B cells and NK cells were analyzed by gating in lymphoid cells. The phenotypes of T cells, including T naïve cells (CD45RA^+^ CCR7^+^), T central memory cells (TCM; CD45RA^−^ CCR7^+^), T effector memory cells (TEM; CD45RA^−^ CCR7^−^) and TEMRA cells (TEMRA; CD45RA^+^ CCR7^−^) populations were analyzed by identifying CD4^+^ T cells and CD8^+^ T cells. Additionally, Tim-3 and PD-1 expression on different T cell phenotypes was examined. Anti-human antibodies were used that targeted the following proteins: CD19 (FITC, HIB19, BD Bioscience), CD56 (APC, B159, BD Bioscience), CD3 (Pacific Blue, UCHT1, BD Bioscience), CD4 (FITC, A161A1, Biolegend), CD8 (PE-cy7, SK1, BD Bioscience), CD45RA (APC, HI100, BD Bioscience), CCR7 (APC-eflour-780, 3D12, eBioscience), Tim-3 (PE, 344823, R&D) and PD-1 (PE, EH12.2H7, Biolegend), as well as isotype control monoclonal antibodies (MABs; BD Bioscience). Whole blood (100 μl) was incubated with MABs for 30 min at 4°C in the dark. Cells were washed once with phosphate-buffered saline (PBS). Detection was performed with a BD FACS Canto II flow cytometer (BD, USA), and the results were analyzed with DIVA software and FlowJo 10.0 software. Isotype-matched immunoglobulins were used as controls.

The concentrations of TNF-α and IL-1β were detected using Human Th1/Th2 Cytometric Bead Array (CBA) Kit II (Becton Dickinson, CA, USA). The concentrations of IFN-γ, IL-10 and IL-6 were detected using a Human Enhanced Sensitivity Flex set (BD Bioscience, San Jose, CA, USA). Detection was performed with the BD FACS Canto II flow cytometer, and the results were analyzed with DIVA software.

### Statistical Analysis

The data analysis was performed using SPSS 18.0 software. The data are presented as the mean ± standard deviation. Descriptive statistics were performed to determine whether the data were normally distributed. Comparisons between groups were performed with one-way analysis of variance (ANOVA), and a *post hoc* test was used to determine differences between groups. Pearson correlation analysis was performed to assess associations between PD-1, TNF-α, IFN-r, IL-6 and IL-10 expression and the absolute number of T cells.

## Results

### Subject Demographics

There were no significant differences in age, education, race, BMI, sex, smoking status, or infection status between the IS groups and the control group. There was a slightly increased degree of alcohol use, hypertension, diabetes mellitus and hypercholesterolemia in the IS groups compared with the control group. The NIHSS scores were significantly decreased in the sub-acute phase group and the stable phase group compared with those in the acute phase group, while the MMSE scores were increased in the sub-acute phase and stable phase groups (*P* < 0.01; Table 1).

**Table 1:** Demographics and baseline characteristics.

Variables of interest	Control group (*n* = 14)	Acute phase group (*n* = 18)	Sub-acute phase group (*n* = 31)	Stable phase group (*n* = 15)
Age (years; mean ± SD)	53.36 ± 7.10	57.50 ± 15.26	59.61 ± 10.42	60.33 ± 6.60
Education (years; mean ± SD)	12.29 ± 2.67	11.67 ± 2.28	11.32 ± 2.29	11.80 ± 2.40
Race, Han, % (*n*)	100	100	100	100
BMI (kg/m^2^; mean ± SD)	22.90 ± 2.01	22.35 ± 2.13	22.19 ± 2.10	22.35 ± 1.92
Sex (% female)	50.00	44.44	41.94	40.00
NISSH score (mean ± SD)	NA	8.89 ± 1.37	4.16 ± 1.24**	2.00 ± 1.17**
MMSE score (mean ± SD)	NA	16.50 ± 6.59	20.42 ± 6.58*	23.50 ± 3.77**
Smoking status (% current smokers)	35.71	50.00	35.48	46.67
Alcohol use status (% current alcohol users)	0.00	41.18	35.48	40.00
Hypertension, % (*n*)	0.00	61.11	61.29	66.67
Diabetes mellitus, % (*n*)	0.00	38.89	38.71	33.33
Hypercholesterolemia, % (*n*)	0.00	50.00	48.39	53.33
Infection, % (*n*)	NA	50.00	54.84	40.00

*Compared with the acute phase group, P < 0.05; **Compared with the acute phase group, P < 0.01.

### Absolute Numbers of T Cells, B Cells and NK Cells in the IS Groups and the Control Group

**Figure 1:**
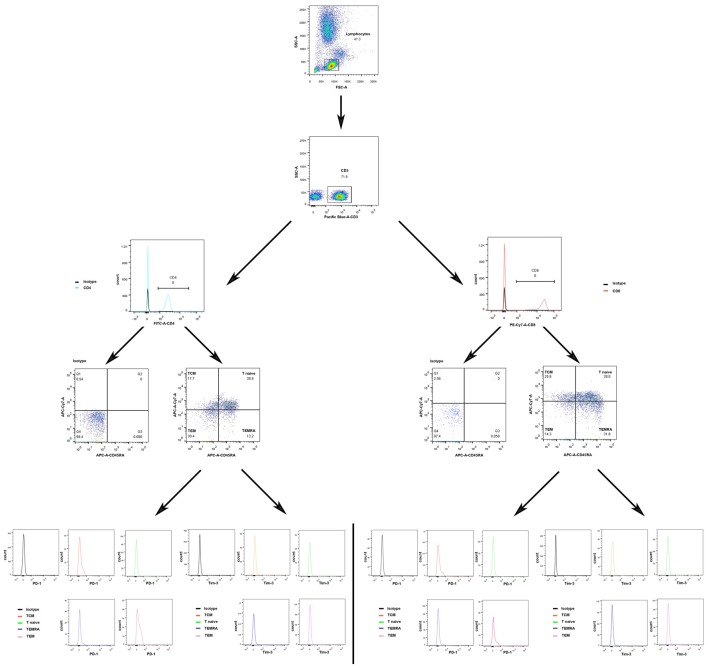
Gating strategies to identify different immune cell populations.

**Figure 2:**
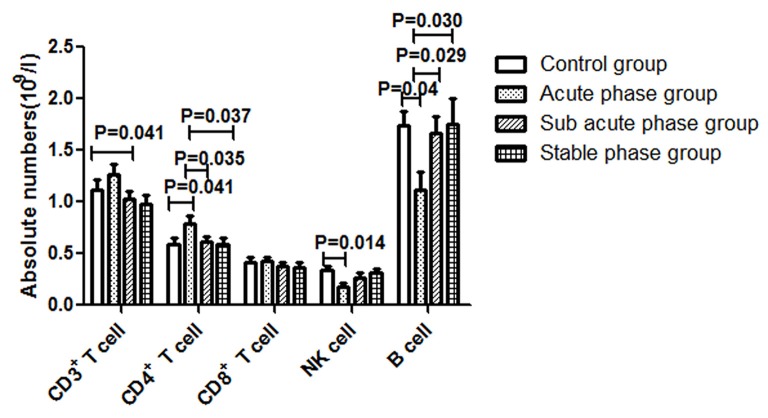
Absolute numbers of T cells, B cells and NK cells in ischemic stroke groups and the control group (comparisons between groups were performed via one-way analysis of variance (ANOVA), and a *post hoc* test was used to determine differences between groups. Graphs show the mean + SEM; Control group (*n* = 14), Acute phase group (*n* = 18), Sub-acute phase group (*n* = 31), Stable phase group (*n* = 15)).

The gating strategies for identification of different immune cell populations are shown in Figure 1. The absolute number of CD4^+^ T cells in the acute phase group was obviously increased compared with that in the control group (*P* < 0.05, Figure 2). The absolute number of CD4^+^ T cells and CD3^+^ T cells was significantly decreased in the sub-acute phase group and the stable phase group, respectively, compared with that in the acute phase group (*P* < 0.05, Figure 2). There were no significant differences in the absolute number of CD8^+^ T cells between any of the IS groups and the control group (*P* > 0.05, Figure 2). The absolute number of NK cells and B cells was significantly decreased in the acute phase group compared with that in the control group (*P* < 0.05, Figure 2). The absolute number of B cells was significantly increased in the sub-acute phase group and the stable phase group compared with that in the acute phase group (*P* < 0.05, Figure 2).

### Quantitative Analysis of Differentiated CD4^+^/CD8^+^ T Cells in the IS Groups and the Control Group

**Figure 3:**
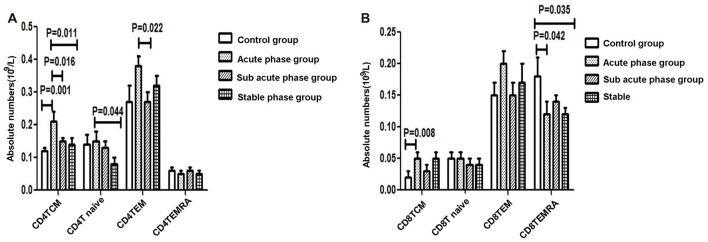
**(A)** Quantitative analysis of differentiated CD4^+^ T cells in ischemic stroke groups and the control group. **(B)** Quantitative analysis of differentiated CD8^+^ T cells in ischemic stroke groups and the control group (comparisons between groups were performed via one-way ANOVA, and a *post hoc* test was used to determine differences between groups. Graphs show the mean + SEM; Control group (*n* = 14), Acute phase group (*n* = 18), Sub-acute phase group (*n* = 31), Stable phase group (*n* = 15)).

The gating strategies used to identify different immune cell populations are shown in Figure 1. A significant increase was noted in the absolute number of CD4^+^ TCM cells in the acute phase group compared with that in the control group, and significant decreases were observed in the absolute numbers of CD4^+^ TCM cells, CD4^+^ TEM cells and CD4^+^ naïve T cells in the sub-acute phase group and stable phase group compared with those in the acute phase group (*P* < 0.05, Figure 3A). The absolute number of CD8^+^ TCM cells in the acute phase group was significantly increased compared with that in the control group. Furthermore, the absolute numbers of CD8^+^ TEMRA cells in the acute phase group and the stable phase group were obviously decreased compared with that in the control group (*P* < 0.05, Figure 3B).

### Cytokine Concentrations in the IS Groups and the Control Group

**Figure 4:**
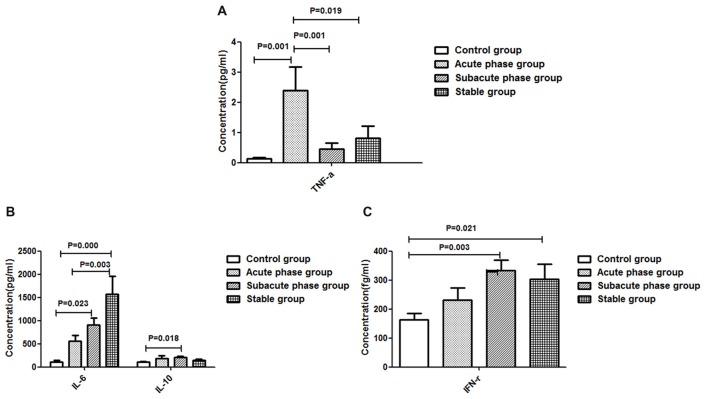
Concentration of cytokines in ischemic stroke groups and the control group. **(A)** Concentration of TNF-α in ischemic stroke groups and the control group. **(B)** Concentration of IL-6 and IL-10 in ischemic stroke groups and the control group. **(C)** Concentration of IFN-γ in ischemic stroke groups and the control group (comparisons between groups were performed via one-way ANOVA, and a *post hoc* test was used to determine differences between groups. Graphs show the mean + SEM; Control group (*n* = 14), Acute phase group (*n* = 18), Sub-acute phase group (*n* = 31), Stable phase group (*n* = 15)).

The TNF-α concentration in the acute phase group showed a significant increase compared with that in the control group (*P* < 0.01, Figure 4A). Additionally, the TNF-α concentrations in the sub-acute phase group and stable phase group were significantly decreased compared with that in the acute phase group (*P* < 0.05, Figure 4A). The IL-10, IL-6 and IFN-r concentrations in the sub-acute phase group were significantly increased compared with those in the control group (*P* < 0.01, Figures 4B,C), and the IL-6 and IFN-r concentrations in the stable phase group were significantly increased relative to that in the control group (*P* < 0.05, Figures 4B,C). However, the IL-1β concentrations were too low to detect.

### Correlations Between Cytokines and the Absolute Number of CD4^+^ T Cells

**Figure 5:**
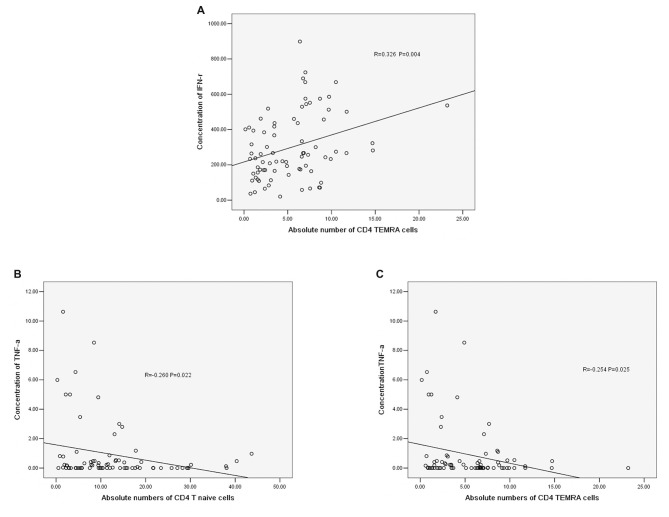
**(A)** Correlation between IFN-r and the absolute number of CD4^+^ TEMRA cells. **(B)** Correlation between TNF-α and the absolute number of CD4^+^ T naïve cells. **(C)** Correlation between TNF-α and the absolute number of CD4^+^ TEMRA cells (correlation determined by Pearson correlation analysis).

The concentration of IFN-r was positively correlated with the absolute number of CD4^+^ TEMRA T cells (*r* = −0.326, *P* = 0.004, Figure 5A). The concentration of TNF-α was negatively correlated with the absolute number of CD4^+^ T naïve cells (*r* = −0.260, *P* = 0.022, Figure 5B) and CD4^+^ TEMRA cells (*r* = −0.254, *P* = 0.025, Figure 5C). However, there was no significant correlation between the concentration of TNF-α or IFN-r and other CD4^+^ T cell phenotypes, and there were no significant correlations between IL-6 and IL-10 concentrations and any of the CD4^+^ T cell phenotypes.

### PD-1 Expression in T Cells in the IS Groups and the Control Group

**Figure 6:**
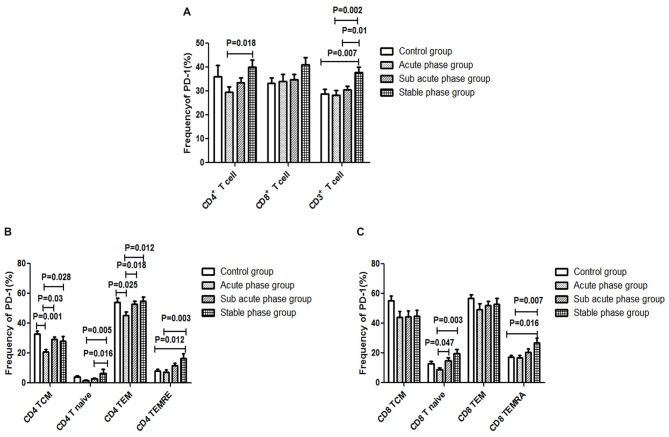
**(A)** Programed cell death-1 (PD-1) expression in T cells in the ischemic stroke (IS) groups and the control group. **(B)** PD-1 expression in CD4^+^ T cells in the IS groups and the control group. **(C)** PD-1 expression in CD8^+^ T cells in the IS groups and the control group (comparisons between groups were performed via one-way ANOVA, and a *post hoc* test was used to determine differences between groups. Graphs show the mean + SEM; Control group (*n* = 14), Acute phase group (*n* = 18), Sub-acute phase group (*n* = 31), Stable phase group (*n* = 15)).

The gating strategies for different immune cell populations are shown in Figure 1. The expression of PD-1 in CD4^+^ T cells and CD3^+^ T cells in the stable phase group showed a significant increase compared with that in the acute phase group (*P* < 0.05, Figure 6A). Particularly, the expression of PD-1 in CD3^+^ T cells in the stable phase group was significantly increased compared with that in the other IS groups and the control group (*P* ≤ 0.01, Figure 6A).

Next, we compared the expression of PD-1 in CD4^+^ T cells and CD8^+^ T cells of different phenotypes. Compared with the control group, the PD-1 expression in CD4^+^ TCM cells and CD4^+^ TEM cells was significantly decreased in the acute phase group (*P* < 0.05, Figure 6B). However, the expression of PD-1 on different CD4^+^ T cell phenotypes in the sub-acute phase group and the stable phase group was significantly increased compared with that in the acute phase group (*P* < 0.05, Figure 6B). The expression of PD-1 on CD8^+^ T naive cells and CD8^+^ TEMRA cells in the sub-acute phase group and the stable phase group was significantly increased compared with that in the acute phase group (*P* < 0.05, Figure 6C).

### Tim-3 Expression in T Cells in the IS Groups and the Control Group

**Figure 7:**
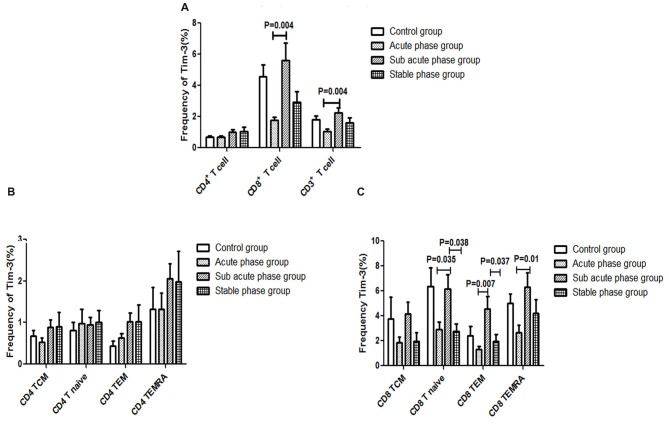
**(A)** T cell immunoglobulin and mucin domain 3 (Tim-3) expression in T cells in the IS groups and the control group. **(B)** Tim-3 expression in CD4^+^ T cells in the IS groups and the control group. **(C)** Tim-3 in expression CD8^+^ T cells in the IS groups and the control group (comparisons between groups were performed via one-way ANOVA, and a *post hoc* test was used to determine differences between groups. Graphs show the mean + SEM; Control group (*n* = 14), Acute phase group (*n* = 18), Sub-acute phase group (*n* = 31), Stable phase group (*n* = 15)).

The gating strategies for different immune cell populations are shown in Figure 1. The expression of Tim-3 in CD8^+^ T cells and CD3^+^ T cells in the sub-acute phase group exhibited a significant increase compared with that in the acute phase group (*P* < 0.01, Figure 7A).

Next, we compared the expression of Tim-3 in CD4^+^ T cells and CD8^+^ T cells of different phenotypes. Our data indicated that there was no significant difference in Tim-3 expression in CD4^+^ T cells of different phenotypes between the IS groups and the control group (Figure 7B). Meanwhile, the expression of Tim-3 in CD8^+^ naïve T cells, CD8^+^ TEM cells and CD8^+^ TEMRA cells in the sub-acute phase group was significantly increased compared with that in the acute phase group (*P* < 0.05, Figure 7C).

### Correlations Between PD-1 and the Absolute Number of CD4^+^ T Cells

**Figure 8:**
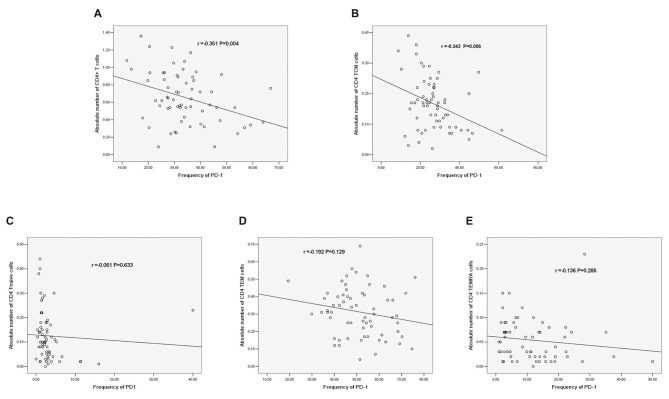
**(A)** Correlation between PD-1 and the absolute number of CD4^+^ T cells. **(B)** Correlation between PD-1 and the absolute number of CD4^+^ TCM cells. **(C)** Correlation between PD-1 and the absolute number of CD4^+^ T naïve cells. **(D)** Correlation between PD-1 and the absolute number of CD4 TEM cells. **(E)** Correlation between PD-1 and the absolute number of CD4^+^ TEMRA cells (correlation determined by Pearson correlation analysis).

There was a negative correlation between PD-1 expression and the absolute number of CD4^+^ T cells (*r* = −0.351, *P* = 0.004, Figure 8A). Additionally, there was a negative correlation between PD-1 and the absolute number of CD4^+^ TCM cells (*r* = −0.343, *P* = 0.006, Figure 8B); however, there was no significant correlation between PD-1 and other CD4^+^ T cell phenotypes (Figures 8C–E).

## Discussion

Increasing evidence indicates that there are significant alterations in the peripheral immune system post-IS. In mice, stroke was reported to induce rapid and long-term cellular immune response suppression (Prass et al., 2003). In humans, cell-mediated immunity is also altered after acute IS (Cunningham et al., 2005; Haeusler et al., 2008). Therefore, T cell dysfunction is considered to play an important role in SIID. However, the mechanism of T cell differentiation during different IS phases is still not well defined. To address this issue, we detected the changes in T cell phenotypes during different IS phases and explored the correlations between PD-1 or Tim-3 and T cell differentiation. Our results showed that the alteration of different T cell phenotypes was involved in the stroke process and that PD-1 may be implicated in the mechanisms of CD4^+^ T cell differentiation in the stroke process.

In our study, we found that the absolute number of peripheral T cells, NK cells and B cells decreased post-stroke, which indicated immune response suppression post-stroke. Additionally, the CD4^+^ T cell number in the acute phase was significantly elevated comparing with that in controls. However, in the sub-acute phase and stable phase, the absolute CD4^+^ T cell number was significantly decreased compared with that in the acute phase. The absolute number of CD3^+^ T cells in the stable phase showed a significant decrease compared with that in the acute phase. These results indicated that peripheral T cells were activated in the acute phase and suppressed in the sub-acute phase and stable phase. The peripheral T cell dysfunction occurred during different IS phases. Additionally, we found that the concentrations of TNF-α, IL-6, IFN-r and IL-10 were significantly increased post-stroke. After the acute phase, the TNF-α concentration decreased, but the IL-6 concentration increased, indicating that the inflammation reactions changed during different stroke phases; in particular, the TNF-α concentration was negatively correlated with the absolute number of CD4^+^ naïve T cells and the absolute number of CD4^+^ TEMRA cells, and the concentration of IFN-r was positively correlated with the absolute number of CD4^+^ T TEMRA cells, which indicated that changes in inflammation reactions accompanied the CD4^+^ T cell alterations.

After antigen priming, naïve T cells trigger antigen-specific T cell responses and differentiate towards effector/memory T cells. TEMRA cells were reported to have a superior capacity to respond to antigenic stimulation than naïve T cells (Champagne et al., 2001; Zippelius et al., 2004). Further studies indicated that TCM cells showed higher proliferation potential and greater resistance to apoptosis. However, TEM/TEMRA cells have a skewed TCR repertoire and a senescent replicative history (Mueller et al., 2001). However, in humans, the relationships among TCM, TEM and TEMRA cells remain unclear.

Klehmet et al. (2016) recently reported that the distribution of T cells changed after stroke, coinciding with the results of our study. However, Klehmet et al. (2016) also found that the naïve T cell population within CD4^+^ T cells and CD8^+^ T cells was reduced early after stroke. In contrast, our results showed that the TCM population in CD4^+^ T cells and CD8^+^ T cells increased early after stroke but decreased after the acute phase of stroke. The difference in the distribution of T cells between our study and the Klehmet et al. (2016) study may be a result of differences in the time points of sample collection.

Certain cell surface proteins, called immune checkpoint receptors (ICRs), have been reported to be associated with T cell function disorder (Blackburn et al., 2009; Wei et al., 2013). Inhibitory molecules, such as PD-1, cytotoxic T lymphocyte associated antigen-4, Tim-3 and killer cell lectinlike receptor G1, were determined to have common features strongly associated with CD8^+^ T cell function exhaustion in viral infection (Barathan et al., 2017). However, the relationship of ICRs with T cells in IS remains unclear, especially during different phases. Therefore, we speculated that Tim-3 and PD-1 play important roles in T cell dysfunction at different phases post-IS.

In this study, we verified our hypothesis and further analyzed the Tim-3 and PD-1 expression in T cells of different phenotypes in different phases post-IS. In addition, we found alterations in PD-1 in CD4^+^ T cells that were contrary to the alterations in the CD4^+^ T cells after the acute phase, which indicated an inhibitory role of PD-1 in CD4^+^ T cell activation. To the best of our knowledge, this is the first report of an inhibitory role of PD-1 in CD4^+^ T cell activation in IS patients after the acute phase. Much has been learned regarding the role of PD-1 on T cells through clinical application of the PD pathway in cancer (Hamid et al., 2013; Zinselmeyer et al., 2013). Bodhankar et al. (2014) confirmed the inhibitory roles of PD-1 and CTLA-4 in T cell activation in a mouse stroke model; however, the role of PD-1 on T cells in IS patients remained unclear. Our study is the first to demonstrate the inhibitory role of PD-1 in CD4^+^ T cell activation in IS patients, which indicates that PD-1 may play an important role during the IS process.

Although the expression of Tim-3 and PD-1 in CD8^+^ T cells changed in different phases, a significant difference in the absolute number of CD8^+^ T cells between IS patients and controls was not observed.

We further analyzed the correlations between PD-1 expression and different CD4^+^ T cell phenotypes. PD-1 was negatively correlated with the absolute number of CD4^+^ T cells (*r* = −0.351, *P* = 0.004). In addition, PD-1 was negatively correlated with the absolute number of CD4 TCM cells among different phenotypes of CD4^+^ T cells (*r* = −0.343, *P* = 0.006). The increased PD-1 expression in CD4^+^ TCM cells may induce the decrease in the absolute number of CD4^+^ T cells. Regarding the role of PD-1 in IS, the conclusion that PD-1 limits the CNS inflammation response and neurologic injury was also reported in a murine model (Bodhankar et al., 2013, 2015); however, few human studies have been reported. Our results are the first to reveal the correlation between PD-1 and T cells in IS patients, and we explored the correlation between PD-1 and T cells in different stroke phases.

In summary, T cell dysfunction, particularly CD4^+^ T cell dysfunction, occurs during different IS phases. We found that PD-1 is negatively correlated with the absolute number of TCM cells among different CD4^+^ T cell phenotypes. Our results preliminarily indicate that PD-1 may play an important role in CD4^+^ T cell dysfunction during the stroke process. Thus, we consider it may be one of the mechanisms underlying SIID, and PD-1 could be a therapeutic target for prevention of infectious complications after stroke. Further study needs to be done to verify the role of PD-1 in stroke.

## Author Contributions

YZ performed the T cell detection, participated in the study design and helped draft the manuscript. LW carried out the cytokine detection experiments and the statistical analysis. YD undertook the sample screening and collection and was involved in statistical analysis of the study results. YX took part in subject collection and the T cell detection. WW and YY designed the study and were involved throughout the entire implementation process.

## Conflict of Interest Statement

The authors declare that the research was conducted in the absence of any commercial or financial relationships that could be construed as a potential conflict of interest. The reviewer SR and the handling editor declared their shared affiliation.
